# Evaluation of Endoscopic Versus Open Lumbar Discectomy: A Multi-Center Retrospective Review Utilizing the American College of Surgeons’ National Surgical Quality Improvement Program (ACS-NSQIP) Database

**DOI:** 10.7759/cureus.25202

**Published:** 2022-05-22

**Authors:** Paul S Page, Simon G Ammanuel, Darnell T Josiah

**Affiliations:** 1 Neurological Surgery, University of Wisconsin, Madison, USA

**Keywords:** microdiscectomy, lumbar discectomy, endoscopic lumbar discectomy, degenerative joint disease, spine

## Abstract

Introduction: Endoscopic techniques in spine surgery continue to gain popularity due to their potential for decreased blood loss and post-operative pain. However, limited studies have evaluated these techniques within the United States. Additionally, given the limited number of practitioners with experience in endoscopy, most current studies are limited by a lack of heterogeneity.

Methods: The American College of Surgeons’ National Surgical Quality Improvement Program (ACS-NSQIP) database was queried to evaluate the effect of endoscopic surgery on adverse events. Current Procedural Terminology (CPT) codes for open discectomy were compared with the relevant CPT codes for endoscopic lumbar discectomy. Baseline patient characteristics and adverse outcomes were then compared.

Results: A total of 38,497 single-level lumbar discectomies were identified and included. Of these, 175 patients undergoing endoscopic discectomy were compared with 38,322 patients undergoing open discectomy. Endoscopic discectomy demonstrated a shorter operative time of 88.6 minutes than 92.1 minutes in the open group. However, this was not significant (p=0.08). Patients in the endoscopic group demonstrated a shorter total length of stay of 0.81 days vs 1.15 days (p=0.014). Total adverse events were lower in the endoscopic group at 0.6% vs 3.4% in the open group (p=0.03).

Conclusion: Endoscopic discectomy demonstrated a significantly lower rate of adverse events and shorter total length of stay than open discectomy. Further research is necessary over time to evaluate larger patient populations as this technology is more rapidly incorporated.

## Introduction

Lumbar radiculopathy is a common clinical pathology with a lifetime incidence ranging from 13% to 40% and is associated with significant morbidity and socioeconomic burden [1]. In cases where conservative therapy fails, discectomy is the standard surgical care. The first lumbar discectomy ever performed was conducted by Fedor Krause in 1908 [2]. Since then, perpetual technologic advancements have pushed the procedure to increasingly more minimally invasive options to reduce postoperative pain and minimize surrounding tissue disruption [3,4].

Most recently, the use of endoscopic technology has become more commonly utilized in the United States. Despite reports of its success and noninferiority abroad, little research comparing endoscopic discectomy compared to traditional open microdiscectomy has been conducted in the United States [5]. Given the relatively small number of practitioners using this technology, large studies comparing outcomes have been difficult to generalize. Here, we evaluated the American College of Surgeons National Surgical Quality Improvement (NSQIP) database to evaluate the nationwide experience of endoscopic discectomy (ED) compared with traditional open discectomy (OD) to evaluate the generalizability of this procedure and its outcomes. Our results are then compared to the known relevant medical literature.

## Materials and methods

A retrospective review was conducted of the ACS-NSQIP database for the years 2017 to 2020 containing data from over 700 hospitals both nationally and internationally [6]. The year 2017 was chosen as it was the first year in which a separate Current Procedural Terminology (CPT) code was available for endoscopic (ED) procedures. Patients were then selected based upon their primary CPT for their procedure being either 62380 representing a fully endoscopic procedure via either an intralaminar or transforaminal approach compared with 63030 which represents an open discectomy (OD) that may or may not include microscopic assistance or use of a tubular retractor device. Following this, patients' demographic characteristics were identified and compared as well as preoperative risk factors including Charlson comorbidity index (CCI), American Society of Anesthetist class (ASA), and wound classification. Primary endpoints included were operative time, length of stay, adverse events, and 30-day readmission rates following procedure. Adverse events were defined as surgical site infections, unplanned intubation, wound complications, renal insufficiency, pneumonia, pulmonary embolism, deep vein thrombosis, urinary tract infections, myocardial infarction, cardiac arrest, blood transfusion, and sepsis.

Statistics were conducted using Statistical Package for Social Sciences (SPSS) version 23 (IBM Corp., Armonk, NY, US). Chi-square was used for categorical variables and unpaired t-tests were performed on continuous variables. Comparisons were made between endoscopic and open procedures for baseline characteristics. Propensity-adjusted multivariate logistic regression was adjusted for age, sex, body mass index, ASA class, and modified CCI. Baseline characteristics and primary endpoints were compared between the prosperity score-matched groups. Statistical significance was considered with a p-value < 0.05.

## Results

Following identification of the relevant CPT codes, 38,497 records were identified as undergoing lumbar discectomy. Of these patients, 38,322 underwent OD with or without the use of a tubular retractor system compared with 175 patients who underwent an ED via either an intra-laminar or transforaminal approach. Patients undergoing ED were significantly older at 53.95 +/- 1.14 compared to patients aged 51.36 +/- 0.08 years old (p=0.025). However, this was not found to be significant following propensity adjustment. No other baseline characteristic variables were found to be significant including BMI, sex, race, CCI, ASA class, or preoperative wound classification (p>0.05), as shown in Table 1.

**Table 1:** Baseline characteristics of patients undergoing decompressive surgery via open or endoscopic techniques ASA: American Society of Anesthetists, BMI: Body Mass Index, CCI: Charlson comorbidity index

Baseline characteristics of patients undergoing decompressive surgery via open or endoscopic techniques			
Variables	Open (N=38,322)	Endoscopic (N=175)	
Age	51.36±0.08	53.95±1.14	
BMI	30.00±0.08	29.4±0.66	
Male Sex	21548 (56.1%)	104 (59.4%)	
White Race	28453 (74.1%)	126 (72.0%)	
Home Discharge	36997 (96.4%)	171 (97.7%)	
CCI			
0	16890 (44.0%)	64 (36.6%)	
1	5112 (13.3%)	21(12.0%)	
2	4243 (11.1%)	22 (12.6%)	
3+	12151 (31.6%)	68 (38.9%)	
Wound Classification			
Clean	38112 (99.3%)	175 (100%)	
Clean-contamined	133 (0.3%)	0 (0%)	
Contamined	31 (0.1%)	0 (0%)	
Dirty	120 (0.3%)	0 (0%)	
ASA Class			
1	3766 (9.8%)	11 (6.3%)	
2	21569 (56.2%)	95 (54.3%)	
3	12496 (32.6%)	67 (38.3%)	
4	525 (1.4%)	2 (1.1%)	
5	4 (0.0%)	0 (0%)	

Following this, patient outcomes were then classified based on their procedure (Table 2). Patients undergoing OD demonstrated a statistically significant longer length of stay than the endoscopic group of 1.15+/-0.02 days compared with 0.81+/-0.12 days (p=0.006). This finding was corroborated by propensity-adjusted analysis (p=0.014). Operative time was found to be shorter compared in ED at 88.6+/-3.23 minutes compared to 92.1+/-0.28 minutes in the OD. However, this result was not statistically significant (p=0.28). Following completion of the procedure, readmission rates were comparable between the two groups with 1221 (3.2%) of those in the open group being readmitted within 30 days compared with 8 (4.6%) in the endoscopic group (p=0.28).

**Table 2:** Comparison of adverse events for open vs endoscopic decompressions CPR: Cardiopulmonary resuscitation

Comparison of adverse events for open vs endoscopic decompressions				
	Open (N=38,322)	Endoscopic (N=175)	p-value	Propensity-adjusted p-value
Adverse Events	1321 (3.4%)	1 (0.6%)	0.03	0.22
Superficial surgical site infection	307 (0.8%)	0 (0.0%)	1.00	0.50
Deep surgical site infection	94 (0.2%)	0 (0.0%)	1.00	
Organ Space surgical site infection	126 (0.3%)	0 (0.0%)	1.00	
Wound dehiscence	73 (0.2%)	1 (0.6%)	0.29	1.00
Pneumonia	70 (0.2%)	0 (0.0%)	1.00	1.00
Unplanned intubation	31 (0.1%)	0 (0.0%)	1.00	
Pulmonary Embolism	87 (0.2%)	0 (0.0%)	1.00	
Ventilator > 48 hours	19 (0.0%)	0 (0.0%)	1.00	
Progressive renal insufficiency	10 (0.0%)	0 (0.0%)	1.00	
Acute renal failure	7 (0.0%)	0 (0.0%)	1.00	
Urinary tract infection	214 (0.6%)	0 (0.0%)	1.00	
Stroke/cerebrovascular accident	21 (0.1%)	0 (0.0%)	1.00	
Cardiac arrest requiring CPR	17 (0.0%)	0 (0.0%)	1.00	
Myocardial infarction	39 (0.1%)	0 (0.0%)	1.00	
Blood transfusion	254 (0.7%)	0 (0.0%)	0.63	0.50
Deep Venous Thrombsis	130 (0.3%)	0 (0.0%)	1.00	
Sepsis	137 (0.4%)	0 (0.0%)	1.00	
Septic Shock	15 (0.0%)	0 (0.0%)	1.00	
30 Day Readmissions	1221 (3.2%)	8 (4.6%)	0.28	1.00

As for complications, the total number of adverse events were found to be significantly less in the ED group occurring in only one case (0.6%), which included one wound dehiscence, compared with 1321 (3.4%) of cases of OD (p=0.03). This result, however, was not corroborated by the propensity-adjusted analysis (p=0.22). No other cases of other complications were found in the ED including surgical site infections, pneumonia, pulmonary embolism, urinary tract infections, or blood transfusions.

## Discussion

Lumbar radiculopathy secondary to a disc herniation is a major cause of morbidity and cost within the United States occurring with a lifetime incidence of 1% to 2% [7]. While discectomy as a surgical procedure was first conducted in 1908, technological advances such as the operative microscope as well as improved retractors and lighting have led to progressively less blood loss and postoperative pain resulting in shorter operative times and hospital length of stay. Endoscopic spine surgery initially garnered interest in 1973 when Parviz Kambin first described the transforaminal endoscopic access route to percutaneously access the disc space [8]. Despite this, it was not until Yeung introduced the first endoscopic spine system in 1993 to perform discectomies under direct visualization that the procedure began to increase in popularity [8,9]. Since this time, the use of endoscopic techniques has gained popularity given their ability to limit surrounding tissue disruption while still achieving the goal of surgical decompression [10]. Given regional trends and socioeconomic factors, the use of ED has largely been championed outside of the United States [11]. Here, we evaluated the ACS-NSQIP database to evaluate national trends in the adoption and use of ED.

The clinical outcomes and efficacy of ED have been evaluated by several studies in comparison with OD. In a recent randomized controlled trial by Gibson et al., 70 patients were randomized to either transforaminal ED or microdiscectomy [12]. In their study, patients undergoing ED demonstrated a statistically significant shorter hospitalization compared with microdiscectomy (0.7 days ± 0.7, range 0-2 vs 1.4 days ± 1.3, range 0-9, p=0.001). As for patient-reported outcome measures, in their series Oswestry disability index (ODI), visual analog scale (VAS), and 36-item short-form survey (SF-36) all improved significantly regardless of surgical procedure, demonstrating equivalence in outcomes (p<0.05). Another recent randomized controlled trial in the Netherlands by Gadijradj et al. evaluated 179 patients undergoing ED compared with 309 OD patients. Patients randomized to ED had a significantly lower VAS for leg pain compared to those in OD (between-group difference of 7.1, 95% confidence interval 2.8 to 11.3) [5]. Additionally in their series, ODI, VAS back pain, and self-perceived recovery all demonstrated equivocal results favoring neither group definitively. These results have also been corroborated by several non-randomized trials including a large retrospective series in China by Hua et al. in 2022 [13].

In routine cases, operative time is frequently an important measure of clinical efficiency as well as cost-efficiency. Several studies have previously been conducted evaluating the operative time of ED compared with OD. In a recent systematic review and meta-analysis conducted by Barber et al., nine studies were identified as comparing outcomes in patients undergoing ED compared with OD. In their series, it was found that operative time was not significantly different. However, there was a trend toward faster operative times with endoscopic discectomy compared with open (mean difference 8.6 minutes, 95% CI -0.62 to 17.81; studies = 9; I2 = 93%; p = 0.07) [14]. Another meta-analysis conducted by Muthu et al. evaluated all ED randomized controlled trials currently available [15]. Despite heterogeneity in their series, a random-effects model was used that demonstrated a significantly shorter operative time in ED compared with OD (63.37 and 73.19 minutes, p=0.23). This result was corroborated by our series which demonstrated a shorter operative time in the ED group compared with the OD group. However, this also was not statistically significant.

Regarding complications, some studies have shown lower rates of complications in patients undergoing ED compared with OD. In the previously discussed meta-analysis by Barber et al., after evaluating eight series in which complications were reported, fewer complications were present in the ED group. However, this finding was not statistically significant (OR 2.12, 95% CI 0.97-4.64; studies = 8; I2 = 35%; p = 0.06) [15]. Another recent systematic review and meta-analysis by Li et al. demonstrated lower rates of complications with ED compared with OD. In their series, ED demonstrated a significantly lower complication rate compared with microdiscectomy (13.4% vs 32.1%, OR: 0.32;P < .001). When evaluated more specifically, surgical site infections routinely demonstrate a significantly lower rate in patients treated endoscopically compared to open discectomy. However, as rates of wound infections are frequently low in microdiscectomy, reaching statistical significance is difficult [16]. Additionally, rates of durotomies are infrequently reported in the current literature due to their very rare occurrence. In one study evaluating 907 patients undergoing ED over four years, only four incidental durotomies (0.4%) were encountered and none of these were direct repairs [17].

Here within, we present a large retrospective review of the ACS-NSQIP database comparing ED to OD. This information assists in assessing the generalizability of ED at the national level to better understand trends in ED utilization and its direct comparison to OD techniques. While these techniques have been reported in large numbers internationally, social factors and national infrastructure may confound results such as length of stay, operative time, and readmission rates [15]. Through the use of national databases, assessing trends in which numerous surgeons are involved is beneficial as opposed to smaller trials where only a few surgeons are included.

Our study does present several limitations. Firstly, while this is one of the largest studies to date and brings generalizability, the overall number of patients undergoing ED is not large and a higher level of evidence trials already exists including several randomized controlled trials. Additionally, our study does not evaluate patient-reported outcomes measures and thus comparing the clinical efficacy of ED compared with OD is impossible. As the role of ED continues to grow in the United States, further evaluation will be necessary to evaluate its adoption and outcomes compared to OD. Additionally, a selection bias is certainly present where patients undergoing endoscopic discectomy are carefully selected as it often occurs under local anesthetic. 

## Conclusions

Endoscopy is a non-inferior option to OD concerning operative time, length of stay, and 30-day readmission, and may offer a lower rate of adverse events. Further research is necessary over time to evaluate larger patient populations as well as surgeon learning curves as this technology is more rapidly incorporated.
